# Association between lower serum vitamin D (25-hydroxy-cholecalciferol) concentrations and cognitive impairment in older adults: data from a populational-based cohort study in a middle-income country

**DOI:** 10.1017/S1368980021004407

**Published:** 2022-09

**Authors:** Luísa Harumi Matsuo, Susana Cararo Confortin, Gilciane Ceolin, Claudia Soar, André Junqueira Xavier, Eleonora D’Orsi, Júlia Dubois Moreira

**Affiliations:** 1 Postgraduate Program in Nutrition, Universidade Federal de Santa Catarina, Florianopolis, Brazil; 2 Department of Collective Health, Universidade Federal do Maranhão, Maranhão, Brazil; 3 Department of Nutrition, Universidade Federal de Santa Catarina, Florianopolis, Brazil; 4 Department of Medicine, Universidade do Sul de Santa Catarina, Florianopolis, Brazil; 5 Postgraduate Program in Public Health, Department of Public Health, Universidade Federal de Santa Catarina, Florianopolis, Brazil; 6 Translational Nutritional Neuroscience Working Group, Postgraduate Program in Nutrition, Department of Nutrition, Universidade do Sul de Santa Catarina, Florianopolis 88040-900, Brazil

**Keywords:** Aged, Aging, Vitamin D, 25-hydroxy-cholecalciferol, Cognitive impairment

## Abstract

**Objective:**

To investigate the association between serum vitamin D (25-hydroxy-cholecalciferol) (25(OH)D) concentrations and cognitive impairment in older adults living in Southern Brazil.

**Design:**

Cross-sectional analysis using data from the second follow-up wave of the populational-based EpiFloripa Aging Cohort Study was collected in 2013–2014.

**Setting:**

Cognitive impairment was evaluated using the Mini-Mental State Examination (MMSE). Blood samples were collected to measure serum vitamin D concentrations using a chemiluminescent microparticle immunoassay. Vitamin D concentrations were distributed in quartiles (Q1: 4·0–20·7 ng/ml; Q2: 20·8–26·6 ng/ml; Q3: 26·7–32·0 ng/ml and Q4: 32·1–60·1 ng/ml), and its association with cognitive impairment was tested by crude and adjusted logistic regression (sociodemographic, behavioural and health aspects) using Q4 as a reference group.

**Participants:**

200 men and 371 women aged 60 years or older participated in this study.

**Results:**

The prevalence of probable cognitive impairment was 21·7 %. Those without cognitive impairment had a higher mean of vitamin D serum concentrations (26·8 *v*. 24·6, *P* = 0·014). In the crude analysis, only individuals in Q2 of vitamin D presented an increased risk for probable cognitive impairment compared with Q4 (highest quartile) (OR 2·65, 95 % CI 1·46, 4·81), remaining significant in the adjusted analysis (OR 6·04, 95 % CI 2·78, 13·13). While Q1 (lowest quartile) was not associated in the crude analysis, but when adjusted, an increased risk of cognitive impairment was observed.

**Conclusion:**

The lowest quartile of vitamin D was directly associated with probable cognitive impairment in older adults in Southern Brazil. More studies are needed to investigate whether maintaining adequate serum levels may represent a significant factor in preventing age-related neurological disorders as well as to verify the need for new cutoff points for this age group.

Demographic transition is a challenging socio-economic aspect in the world, especially in low- and middle-income countries. Individuals aged 60 or over represent 12 % of the world’s population, which is estimated to reach 22 % in 2050^(1)^. Populational aging is one factor responsible for the changes in the morbidity and mortality profile in the population, such as the increased prevalence of chronic non-communicable diseases, including mental and neurological disorders^(2)^. Mental and neurological disorders affect more than 20 % of the older adult population worldwide, and dementia is among the most common disorders, affecting approximately 5 % of this age group^(3)^. According to the World Alzheimer Report 2019, it is estimated that there are more than 50 million people in the world with some type of cognitive impairment, and the number is expected to exceed 152 million by 2050^(4)^. In low- and middle-income countries, the number of people suffering from dementia is increasing rapidly with age^(4)^. In addition to the impact on health, mental problems have significant economic repercussions. In 2019, it is estimated that the annual cost of mental disorders worldwide reached 1 trillion dollars^(5)^.

Understanding the risk factors for impaired cognitive function allows for the targeting of interventions to prolong autonomy or delay the onset of dementia^(6)^. Evidence suggesting a relationship between vitamin D and brain development, neurotransmission, neuroprotection and immunomodulation has been growing^(7)^. In epidemiological studies, low levels of vitamin D in older adults have been associated with poorer cognitive performance^(8,9)^. The maintenance of adequate levels of vitamin D could, therefore, represent an important protective factor in the prevention of neurological disorders related to aging. It is postulated that vitamin D deficiency represents a global health problem^(10)^. In Brazil, despite high ultraviolet radiation availability to produce vitamin D in the skin throughout the year, studies have indicated a high prevalence of insufficiency and deficiency^(11–14)^, not supporting the common assumption that the level of radiation solar energy in the country guarantees adequate levels of vitamin D^(15)^. As people age, the risk of vitamin D deficiency increases significantly, mainly due to the decreased capacity for synthesis in the skin^(16,17)^. In addition, older adults are among the risk groups for vitamin D deficiency which has been related to less sun exposure, decrease in food intake with Vitamin D and intestinal malabsorption^(18,19)^.

In low- and middle-income countries, evidence on the association between vitamin D and cognitive impairment in the older population is scarce^(20,21)^. Therefore, the objective of this study was to investigate the association between serum vitamin D (25-hydroxy-cholecalciferol) concentrations and cognitive impairment in the older population in the southern region of Brazil.

## Materials and methods

### Study design and population

**Fig. 1:**
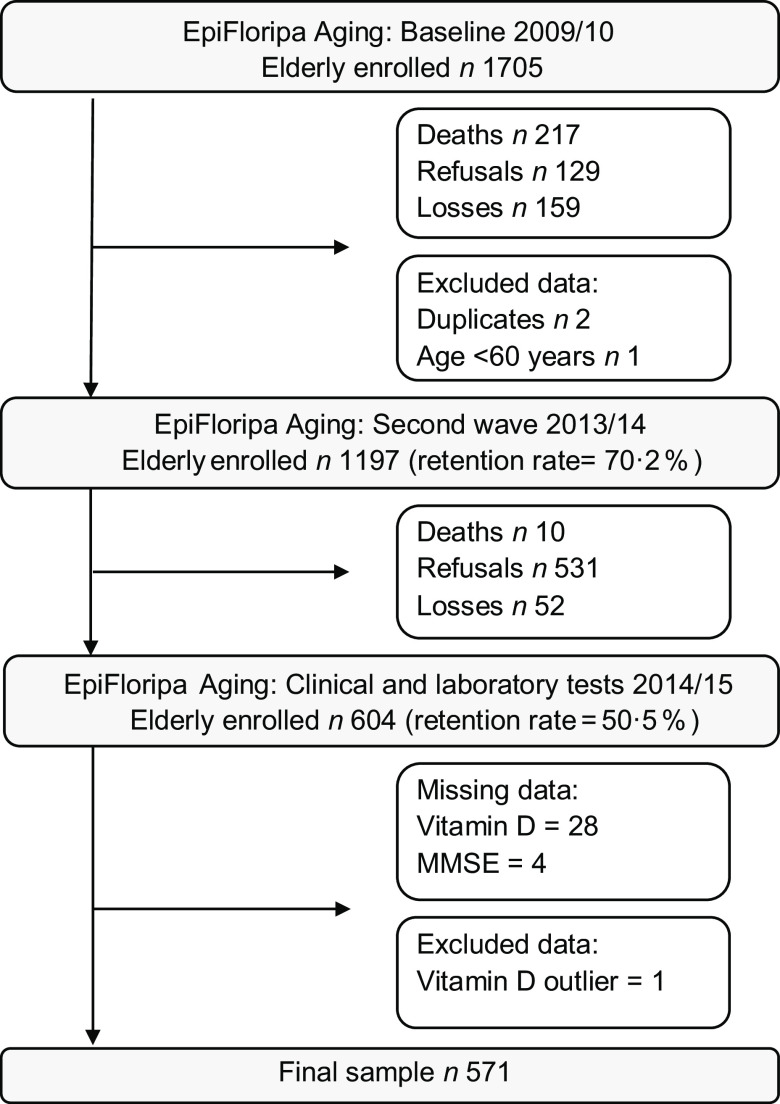
Flowchart of the populational-based EpiFloripa cohort study

This is a cross-sectional analysis of data collected in 2013–2014 from the database of household populational-based EpiFloripa Aging Cohort Study (www.epifloripa.ufsc.br). The EpiFloripa Aging Study design and methods have been previously published^(22,23)^. EpiFloripa’s baseline was established in 2009/2010 with a sample of 1702 older adults living in *Florianópolis* city, *Santa Catarina* State, Southern Brazil (Fig. 1). Older adults of both sexes, aged 60 years or more at the time of the interview, living in the sectors sampled by the survey were considered eligible at baseline. Older adults who were institutionalised (living in long-term care institutions, hospitals, prisons) were excluded. In the follow-up, carried out in 2013/2014, everyone who participated in the EpiFloripa baseline were included, resulting in 1197 interviews (response rate of 70·2 % in relation to the baseline)^(23)^. All the older adults in the follow-up were invited to provide blood samples for analysis of biochemical markers, including vitamin D (*n* 604, response rate of 50·5 %)^(24)^. The average interval between interviews and blood collection was 107 d (with a median, 25th and 75th percentile of this difference of 100, 45 and 136 d, respectively). In this study, 572 older adults who participated in the second follow-up wave with complete data to assess cognitive impairment and vitamin D were included in the analysis. One outlier of serum vitamin D was excluded from the analysis (>96 ng/ml without supplementation), resulting in an analytical sample of 571 individuals. The interviews were conducted by trained interviewers at the older adult’s home, with the help of laptops. To ensure quality control, the use of validated instruments was prioritised for the composition of the questionnaire as well as the selection of interviewers with training in the health area and experience in research. Quality control was performed by telephone, using a short version of the questionnaire in 10 % of the sample.

### Cognitive assessment

The cognition was assessed using the Mini-Mental State Examination (MMSE), translated and validated in Brazil by Bertolucci *et al.* (1994)^(25)^. The MMSE is one of the most widely used screening tools to identify cognitive impairment in clinical practice^(26,27)^. The scores were categorised as probable cognitive impairment and absence of cognitive impairment, considering schooling of older adults, using the cutoff points 19/20 for illiterate and 23/24 for any level of education, as recommended by Almeida (1998)^(28)^.

### Serum vitamin D (25-hydroxy-cholecalciferol)

For the measurement of 25-hydroxy-cholecalciferol (25(OH)D), individuals were invited to attend the Laboratory for Metabolism and Dietetics in the university between 7 and 10 a.m. Blood samples were collected after an 8-hour fast by venipuncture^(24)^. Blood samples were processed and analysed by the clinical analysis laboratory of the University Hospital of the Federal University of Santa Catarina. Serum 25(OH)D concentrations were measured using the Microparticle Chemiluminescence method/LIAISON, which is considered a rapid, accurate and accurate assay (Functional sensitivity: ≤ 2·0 ng/ml; inter-assay inaccuracy < 20 %)^(29,30)^. The LIAISON® 25OH vitamin D assay (Diasorin, São Paulo, Brazil) is certified of total 25-hydroxyvitamin D assays by CDC vitamin D Standardization-Certification Program (CDC VDSCP) since 2014^(31)^. First, the blood samples were centrifuged (3500 rpm) for 10 min and the serum samples were immediately processed using the LIASON® according to the manufacturer^(30)^. Briefly, an antibody specific to vitamin D was coated on magnetic particles, and 25(OH)D conjugated to an isoluminol derivative and diluted in phosphate buffer (pH 7·4). In the first incubation period, 25-OHD dissociated from the binding protein, and it interacts with the antibody. After the second incubation with the tracer reagent, microplate is washed with the buffer and starter reagents are added to generate the chemiluminescent signal, which is measured by a photomultiplier.

After that, serum concentration of vitamin D was divided into quartiles (Q1: 4·0–20·7 ng/ml; Q2: 20·8–26·6 ng/ml; Q3: 26·7–32·0 ng/ml; and Q4: 32·1–60·1 ng/ml) for statistical analysis.

### Covariates

To characterise the sample and adjust the analysis, socioeconomic, demographic, behavioural and health aspects were categorised as follows: gender (male and female); age (63–69 years, 70–79 years or 80 years or more); schooling (0–4 years, 5–11 years or 12 years or more); per capita income in minimum wages (MW) according to the values in 2013 and 2014 (≤ 3 MW > 3, and ≤ 5 MW > 5); marital status (married, single/divorced, or widowed), smoking (no, past, or currently smoker); alcohol consumption (never, moderate (consumes up to one dose of alcohol on an average day and never consumes 5 or more doses on a single occasion), or high (consumes more than 2 doses in one a normal day or 5 or more doses on a single occasion)) according to the Alcohol Use Disorder Identification Test^(32)^; leisure-time physical activity (sufficiently active (≥150 min/week), and insufficiently active (<150 min/week)), according to the International Physical Activity Questionnaire^(33,34)^; number of morbidities diagnosed (none, 1, 2 or more (to include the following diseases: arthritis, cancer, diabetes, bronchitis, kidney disease, tuberculosis, cirrhosis, heart or CVD, stroke or cerebral ischemia, osteoporosis, hypertension/high blood pressure, depression))^(35)^; BMI^(36)^ (underweight (<22 kg/m^2^), normal weight (22–27 kg/m^2^) and overweight (≥28 kg/m^2^))^(37)^; season at time of blood collection (spring/summer, autumn/winter); vitamin D supplement use (yes/no). Supplementation of vitamin D was verified by medical prescription and verification of the presence of the supplement in the participant’s residence and self-reported use of vitamin D, registered in the EpiFloripa database according to the WHO Collaborating Center for Drug Statistic Methodology.

### Statistical analysis

The sample was described according to socioeconomic, demographic, behavioural and health characteristics using absolute and relative frequencies, 95 % CI or mean and sd. The difference between probable and absent cognitive impairment was determined by Pearson’s Chi-square test and Fisher’s exact test. The normality of the vitamin D variable was verified by the Shapiro–Wilk test, and the outliers were identified by the boxplot. The difference in serum vitamin D concentrations between supplemented and not supplemented individuals and cognitive status was evaluated using Student’s *t* test and ANOVA followed by Bonferroni post hoc.

To explore the association between cognitive impairment (dependent variable) and vitamin D in quartiles (independent variable), logistic regression was used with crude and adjusted analysis. The adjusted analysis was initiated by socioeconomic and demographic variables (Model 1: gender, age group, income, season and vitamin D supplementation), followed by the inclusion of behavioural variables (Model 2: model 1 + physical activity, smoking and alcohol consumption) and health (Model 3: model 2 + number of morbidities and BMI). The analysis was performed considering Q4 (higher vitamin D levels) as the reference group to investigate the risk associated with vitamin D deficiency. The season during which the blood was collected was used to adjust for seasonal effects, considering that vitamin D levels are partially dependent on exposure to sunlight. Schooling was not included in the adjusted analysis, as it is already used in the classification criteria of the MMSE. The results were presented as OR with their respective 95 % CI. The value of *P* < 0·05 in the Wald test was used for making statistical decisions. To assess the adequacy of fit of the multiple logistic regression model, the Hosmer–Lemeshow test was used^(38)^. Statistical analysis was performed using the STATA® statistical program (Stata Corporation) version 14.0. The effect of the sample design by clusters was considered, incorporating the sample weight using the *svy* command. Statistical significance was set at *P* < 0·05.

## Results

Of the 604 participants in the 2013–2014 wave, 571 older adults met the criteria and presented complete data to be included in the analysis (Fig. 1). Sixty-two percent were women (*n* = 371), and the mean age was 72·3 (sd ± 6·4) years. The prevalence of probable cognitive impairment in the total sample was 21·7 % (Table 1).

**Table 1:** Socioeconomic, demographic and behavioural characteristics of the elderly sample of the study according to the presence of cognitive impairment, EpiFloripa Aging cohort study, follow-up wave 2013–2014, Southern Brazil

Characteristics (*n* = 571)	Total			Absent cognitive impairment			Probable cognitive impairment			*P*
	*n*	%	95 % CI	*n*	%	95 % CI	*n*	%	95 % CI	
Gender										0·171*
Men	200	37·3	32·3, 42·5	163	83·9	75·1, 90·0	37	16·1	10·0, 24·9	
Women	371	62·7	57·5, 67·7	284	75·0	68·6, 80·5	87	25·0	19·5, 31·4	
Age range (years)										< 0·001†
63–69	242	42·3	36·6, 48·2	204	84·1	75·9, 89·8	38	15·9	10·2, 24·1	
70–79	237	41·4	36·2, 46·7	188	80·0	73·1, 85·4	49	20·0	14·6, 26·9	
≥ 80	92	16·3	12·9, 20·5	55	59·2	47·3, 70·0	37	40·8	29·9, 52·7	
Schooling (years)										< 0·001‡
0–4	242	39·6	32·8, 46·7	143	58·5	50·6, 65·9	99	41·5	34·1, 49·4	
5–11	191	37·0	31·9, 42·4	167	86·3	78·8, 91·5	24	13·7	8·5, 21·2	
≥ 12	138	23·4	18·7, 28·9	137	99·2	94·8, 99·9	1	0·7	0·1, 5·2	
Per capita income										< 0·001*
≤ 3 MW	202	37·2	32·1, 42·6	133	66·8	58·2, 74·4	69	33·2	25·6, 41·8	
> 3 and ≤ 5 MW	111	17·2	13·0, 22·4	86	74·8	62·7, 84·0	25	25·2	16·0, 37·3	
> 5 MW	238	45·6	39·1, 52·3	213	90·7	83·9, 94·8	25	9·3	5·2, 16·1	
Marital status										0·016*
Married	323	56·3	50·7, 61·7	266	81·9	74·5, 87·5	57	18·1	12·5, 25·5	
Single/divorce	82	15·9	12·2, 20·5	63	77·6	64·0, 87·0	19	22·4	13·0, 36·0	
Widowed	166	27·9	22·9, 33·4	118	71·6	61·6, 79·8	48	28·4	20·2, 38·4	

Characteristics (*n* 571)	Total			Absent cognitive impairment			Probable cognitive impairment			*P*
	*n*	%	95 % CI	*n*	%	95 % CI	*n*	%	95 % CI	
Smoking										0·551*
Never	362	58·9	53·0, 64·5	279	75·3	68·0, 81·3	83	24·7	18·7, 32·0	
Past	170	33·9	28·9, 39·3	138	83·5	76·1, 88·9	32	16·5	11·1, 23·9	
Currently smoker	39	7·2	5·0, 10·3	30	79·1	62·5, 89·5	9	20·9	10·5, 37·5	
Alcohol (AUDIT)										< 0·001*
Never	330	56·4	50·2, 62·5	233	71·1	63·3, 77·8	97	28·9	22·2, 36·7	
Moderate/high	241	43·6	37·5, 49·8	214	87·7	81·1, 92·2	27	12·3	7·8, 18·9	
Physical activity (IPAC)										< 0·001*
Sufficiently active	170	30·0	24·7, 36·0	152	89·5	83·8, 93·4	18	10·5	6·6, 16·2	
Insufficient active	400	70·0	64·0, 75·3	295	73·6	66·5, 79·6	105	26·4	20·4, 33·5	
Morbidities (number)										0·286*
None	52	9·7	6·9, 13·3	43	85·2	68·2, 93·9	9	14·8	6·1, 31·8	
1	119	21·1	17·4, 25·3	98	81·7	69·8, (89·6	21	18·3	10·4, 30·2	
≥ 2	400	69·3	64·7, 73·4	306	76·3	70·2, 81·5	94	23·7	18·5, 29·8	
BMI (NSI. 2002)										0·003*
Low body mass	48	8·4	6·0, 11·8	32	76·9	62·1, 87·1	16	23·1	12·9, 37·9	
Eutrophic	259	44·2	39·6, 49·1	195	75·3	66·3, 82·6	64	24·7	17·4, 33·7	
Excess body mass	257	47·3	41·7, 53·0	218	82·7	75·7, 88·1	39	17·3	11·9, 24·3	
Vitamin D ng/ml										
Mean	571	26·3		447	26·8		124	24·6		0·014§
sd		8 6			8·5			8·8		
Serum vitamin D (quartiles)										0·036*
Q1 (4·0–20·7 ng/ml)	146	25·4	19·9, 31·8	108	77·4	67·2, 85·1	38	22·4	14·9, 32·8	
Q2 (20·8–26·6 ng/ml)	140	22·2	18·2, 26·8	102	69·4	57·9, 79·0	38	30·6	21·0, 42·1	
Q3 (26·7–32·0 ng/ml)	145	26·1	22·1, 30·5	118	79·3	72·2, 85·0	27	20·7	15·0, 27·8	
Q4 (32·1–60·1 ng/ml)	140	26·3	21·3, 32·1	119	85·7	76·7, 91·7	21	14·3	8·3, 23·3	
Vitamin D supplementation										
No	454	90·5	86·7, 93·3	361	79·9	73·6, 85·1	93	20·1	14·9, 26·4	0·171*
Yes	55	9·5	6·7, 13·3	48	82·8	66·6, 92·1	7	17·2	7·9, 33·4	
Season										
Spring/summer	149	26·2	20·4, 33·1	108	74·6	65·7, 81·8	41	25·4	18·2, 34·3	0·046*
Autumn/winter	422	73·8	66·9, 79·6	339	79·7	73·8, 84·5	83	20·3	15·5, 26·2	

AUDIT, Alcohol Use Disorder Identification Test; IPAQ, International Physical Activity Questionnaire; MW, minimum wages; NSI, Nutrition Screening Initiative.

* Chi-square test.

† Trend chi-square.

‡ Fisher’s exact test.

§ Student *t* test.

Regarding the characteristics of the sample according to the presence and absence of probable cognitive impairment, there was a difference for almost all aspects, except for gender (*P* = 0·171), smoking (*P* = 0·551) and the number of morbidities (*P* = 0·286). The prevalence of cognitive impairment was higher among the older adults over 80 years of age (*P* < 0·001), with an income of up to 3 MW (*P* < 0·001), with less than 4 years of schooling (< 0·001), who are widowed (*P* = 0·0016), who never consumed alcohol (*P* < 0·001), who are physically inactive (*P* < 0·001) and with eutrophic BMI (*P* = 0·003). Regarding vitamin D, there was a lower prevalence of cognitive impairment in the highest vitamin D quartile (*P* = 0·036), and the mean vitamin D serum concentration was higher in those without cognitive impairment (26·8 *v*. 24·6, *P* = 0·014) (Table 1). The prevalence of vitamin D supplement use was low (*n* 55, 9·6 %) for the total sample, and among these, 48 (87·3 %) individuals did not present probable cognitive impairment. The prevalence of cognitive impairment was lower among the older adults supplementing vitamin D, but without a statistically significant difference between the groups (*P* = 0·171) (Table 1).

Regarding vitamin D serum concentrations, the results showed a high prevalence of inadequate levels, considering that 47·6 % of older adults were in Q1 and Q2, with levels between 4·0 and 26·6 ng/ml. The prevalence of vitamin D deficiency and insufficiency in the sample according to Endocrine Society cutoff points was 22·6 and 42·3 %, respectively, and according to Institute of Medicine (IOM) cutoff points was 4·7 % e 17·9 %, respectively (Supplementary Table).

**Fig. 2:**
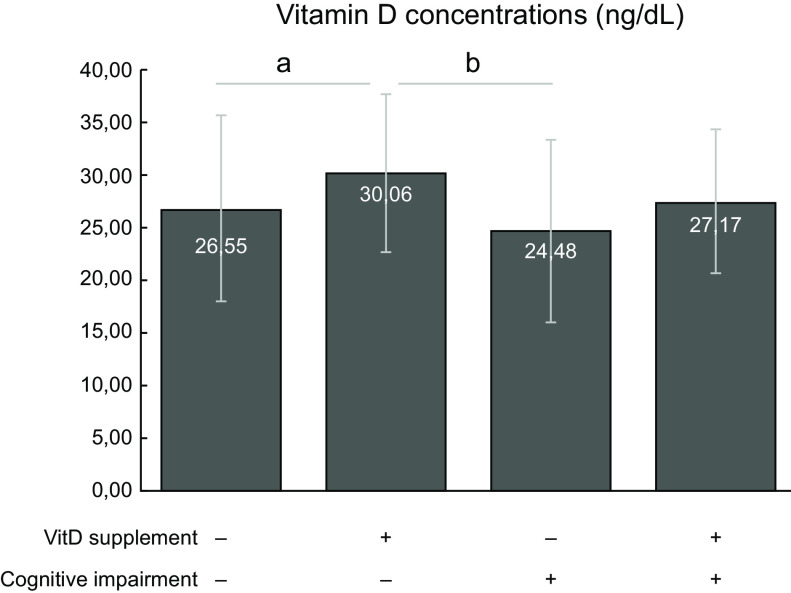
Serum vitamin D concentrations in elderly individuals in relation to supplementation of vitamin D (VitD) and probable presence of cognitive impairment. Data are presented as mean and SD. One-way ANOVA followed by Bonferroni post hoc was used (^a^ *P* < 0·05 between the elderly without cognitive impairment, whether with supplementation or not; ^b^ *P* < 0·05 between older adults with cognitive impairment without supplementation and older adults without cognitive impairment with supplementation)

Using Student’s *t* test, mean serum vitamin D concentrations were found to be higher in vitamin D-supplemented individuals than in those not supplemented (supplemented: mean 29·7, sd ± 7·3, 95 % CI 27·7, 31·7; not supplemented: mean 26·1 sd ± 8·7, 95 % CI 24·3, 26·9; *P* = 0·004). When vitamin D supplementation and cognitive impairment were evaluated together, using ANOVA followed by Bonferroni post hoc, statistical differences were observed for supplemented *v*. not supplemented groups (Fig. 2). Those individuals without cognitive impairment and taking vitamin D supplementation presented higher levels of vitamin D than those without cognitive impairment and without supplementation. There was a difference in the serum vitamin D concentrations between those with cognitive impairment without supplementation and those without cognitive impairment with supplementation.

Logistic regression analysis investigating the association between serum vitamin D level and cognitive impairment are presented in Table 2. In the crude analysis, older adults in Q2 for vitamin D had a higher OR for probable cognitive impairment when compared with adults in Q4, the reference group (OR 2·65, 95 % CI 1·46, 4·81). This association remained statistically significant after the adjustment models. It was observed that individuals in Q1 for serum vitamin D presented an increased risk of probable cognitive impairment in models 1 and 2 (Model 1: OR 2·71, 95 % CI 1·22, 6·04; Model 2: OR 2·72, 95 % CI 1·19, 6·22). In model 3, all individuals below Q4 for serum vitamin D presented an increased risk of probable cognitive impairment (Q1: OR 3·03, 95 % CI 1·37, 6·73; Q2: OR 6·04, 95 % CI 2·78, 13·13; Q3: OR 2·68, 95 % CI 1·18, 6·08). The adjustment of the multivariate logistic regression model was considered adequate by the Hosmer–Lemeshow test (*P* = 0·9575)^(38)^.

**Table 2:** Crude and adjusted logistic regression to investigate the risk for cognitive impairment according to serum vitamin D concentrations in elderly, EpiFloripa Aging cohort study, follow-up wave 2013–2014, southern Brazil

	Crude analysis			Model 1*			Model 2†			Model 3‡,§		
	OR	95 % CI	*P*	OR	95 % CI	*P*	OR	95 % CI	*P*	OR	95 % CI	*P*
Serum vitamin D (quartiles)			0·038			0·007			0·011			0·003
Q4 (32·1–60·1 ng/ml)	1·00			1·00			1·00			1·00		
Q3 (26·7–32·0 ng/ml)	1·57	0·78, 3·14		2·20	1·00, 4·83		2·38	1·00, 5·67		2·68	1·18, 6·08	
Q2 (20·8–26·6 ng/ml)	2·65	1·46, 4·81		5·14	2·49, 10·61		5·37	2·38, 12·11		6·04	2·78, 13·13	
Q1 (4·0–20·7 ng/ml)	1·76	0·87, 3·56		2·71	1·22, 6·04		2·72	1·19, 6·22		3·03	1·37, 6·73	

* Model 1: Gender, age, per capita income, marital status, season, vitamin D supplementation.

† Model 2: Model 1 + smoking, alcohol, physical activity.

‡ Model 3: Model 2 + morbidities, BMI.

§ Hosmer–Lemeshow fit test *P* = 0·9575.

## Discussion

The main finding of this study is the association between lower concentrations of serum vitamin D and cognitive impairment independent of vitamin D supplementation and season, a statistically significant result even after adjusting for potential confounding factors. This is a truly relevant finding, especially because the EpiFloripa Aging study^(23)^ design included a representative population from the city where data were collected in one of the most longevous cities in southern Brazil.

The prevalence of probable cognitive impairment in the total sample was 21·7 %, with 25·0 % for women and 16·1 % for men. Worldwide, there is a great variety in the prevalence of cognitive impairment, with 13·6 % in UK^(39)^, 18·7 % in Singapore^(40)^, 22·2 % in the USA^(41)^ and 23·3 % in Spain^(42)^. In middle-income countries, the prevalence ranged from 7·3 % to 24·1 %^(43)^. The prevalence of cognitive impairment could vary according to the rating scale used as well as the cutoff points for the MMSE applied in the study. Many articles found in the literature evaluating cognitive impairment in older adults used the MMSE as a rating scale^(44–46)^.

Our data have shown that the prevalence of probable cognitive impairment was different for women and men, but without statistical significance. Women accounted for the majority of the sample in our study. Regarding the difference between genders, studies have shown varying results in older adults^(42,47,48)^. However, the higher prevalence among women observed could be due to the education profile, in which men were more likely to have 12 years or more of schooling, and women with 1–4 years of schooling (data not shown). Years of schooling is one of the main factors associated with MMSE performance^(49)^ and cognitive impairment^(50–52)^. We also found differences in the prevalence of cognitive impairment according to marital status. Previous studies found that divorced and widowed elderly people are more vulnerable to cognitive impairment^(53,54)^ and this association is stronger for men than women^(55,56)^. It is suggested that being married is associated with a reduced risk of dementia than lifelong single and widowed^(57)^.

According to the Endocrine Society cutoff point, more than half of the elderly have inadequate levels of vitamin D, but this number is lower with the IOM definition. The 2nd International Conference on Controversies in Vitamin D discussed the cutoff points, recommending that 25(OH)D values below 12 ng/ml should be considered associated with an increased risk of rickets/osteomalacia, while 25(OH)D concentrations between 20 and 50 ng/ml seem to be safe and sufficient for bone health in the general population^(58)^. The Brazilian Society of Endocrinology and Metabolism recommends values between 30 and 60 ng/ml for populations at risk, which includes older adults^(59,60)^. Vitamin D deficiency below 20 ng/ml has been discussed as a risk factor for several health conditions and unfavorable skeletal outcomes, especially in older adults including fractures and bone loss, muscle function and risk of fall and the increased risk of mortality^(61–64)^.

In Brazil, due to its high solar radiation throughout the year, an adequate concentration of serum vitamin D in the population is expected. However, the results found in the literature^(65–67)^ suggested that deficiency is common among older adults. It is important to note that only 26·2% of the blood collections in this study were performed in the spring and summer, the seasons in which the incidence of sunlight is highest^(10)^, and 73·8% in the autumn and winter, when there is a higher prevalence of vitamin D deficiency^(11,42,67)^, which may result in lower serum concentrations of this vitamin. Nevertheless, even when we controlled the analysis for the season, the association was sustained. In addition to the season, other factors also contribute to vitamin D deficiency/insufficiency in older adults, such as female sex, dark skin pigmentation, reduced intake, increased adiposity, shorter outdoor activities, decreased absorption, reduced renal function and medication use^(17)^. Our sample was composed mainly of women, people with insufficient physical activity, low per capita income and few years of schooling, which could influence food acquisition and information about vitamin D-rich foods and the acquisition of vitamin D supplements as well. In our sample, only 9·5 % of the elderly were receiving vitamin D supplements and had higher levels of vitamin D when compared with those who were not supplementing. With these data, we postulate that older individuals are a risk group for vitamin D deficiency and an effort of physicians, gerontologists and nutritionists to investigate this aspect and provide adequate supplementation earlier for the older adult population is needed.

The association between serum vitamin D concentrations and cognitive impairment found in our study is in agreement with previous evidence that analysed vitamin D in quartiles^(20,39,68)^. In UK, a study with a representative sample of 1766 older people investigated the association of vitamin D with cognitive impairment, assessed by the Abbreviated Mental Test. The older adults in the first quartile of vitamin D (3·2–12·0 ng/ml) (OR 2·3; 95 % CI 1·4, 3·8) presented a greater chance of cognitive impairment when compared to those in the highest quartile (26·4 at 68·0 ng/ml). The results suggest that low serum vitamin D levels are associated with increased chances of cognitive impairment^(39)^. In a study with 644 older Japanese adults, using the highest quartile of vitamin D as a reference, those in the lowest quartile had almost 3 times more chance of cognitive impairment (OR 2·70; 95 % CI 1·38, 5·28), defined as a score less than or equal to 23 on the MMSE^(68)^. In China, a study investigated this association in older population and found lower plasma vitamin D levels in individuals with cognitive impairment (score less than 18 in the MMSE) (12·76 ± 6·12 ng/ml) than in those without (18·24 ± 7·84 ng/ml). Older adults in the lowest quartile of vitamin D were twice as likely to have cognitive impairment when compared to those in the highest quartile (OR 2·15; 95 % CI 1·05, 4·41)^(20)^. In 3325 elderly Americans, the adjusted logistic regression model showed that older adults who had severe vitamin D deficiency were more likely to have cognitive impairment (OR 3·68; 95 % CI 1·37, 9·90) compared to those with sufficient serum concentrations^(69)^.

The results of this study are in agreement with those observed in a systematic review with meta-analysis, in which lower concentrations of vitamin D were associated with worse cognitive performance in the older adult population (OR 1·24; 95 % CI 1·14, 1·35). However, the authors report important methodological limitations, such as the heterogeneity of the populations studied, the different forms of cognitive assessment, the different definitions of vitamin D deficiency and uncontrolled confounding factors^(8)^, such as season, which we inserted in our models for the analysis.

Other studies that assessed global cognitive function by MMSE found conflicting findings. In 118 elderly Europeans, a significant association was found with vitamin D tertiles^(70)^. In Norway, a study of 2044 older adults also found no significant relationship between the vitamin D quartiles and the MMES score^(71)^. This association was also not found in a sample of 965 older American adults^(72)^. In this way, studies investigating the association between serum vitamin D concentrations and cognition have found conflicting results. The variety of instruments used to assess the cognitive status and the different cutoff points to define vitamin D deficiency are limitations of these investigations^(73)^.

The putative mechanisms by which vitamin D modulates cognitive processes in aging and the neurophysiopathology of dementia are complex and not well established. Its role in the brain is mediated by the presence of nuclear vitamin D receptors in neurons and glial cells^(74,75)^. The location of these receptors in the hippocampus, hypothalamus, cortex and cerebral subcortex supports the hypothesis of their action in the regulation of neurocognitive functions^(75–77)^. Although vitamin D cutoff points are well defined for the proper maintenance of bone metabolism^(78)^, the ideal concentrations to maintain cognitive function have not yet been identified^(21)^. To maximise its effects on body tissues other than bone mass, it has been suggested that vitamin D concentrations should be in the range of 28–40 ng/ml^(79)^. Thus, we verified the importance of more studies to elucidate new cutoff points for vitamin D for the maintenance of cognitive function in the older population.

This study has some limitations. The response rate to perform laboratory tests (response rate of 50·4 %) is the most important. This was due to the need to attend the exams, which can trigger a selection bias. The participation of older adults with better health conditions could result in underestimating the prevalence of probable cognitive impairment. Because they are walking and are subject to greater exposure to sunlight, the results of vitamin D may also have been overestimated in this sample, not being representative of the general population. A previous study in the EpiFloripa sample compared the refuses and losses with enrolled people and found differences in age (*P* < 0·001) (82·1 % in ≥ 80 years) and the probable presence of cognitive deficit (68·7 %). It was noticed that, with increasing age, there was a reduction in participation in the exams; this reduction was also noted in those with probable cognitive impairment^(24)^. In addition, potential confounding factors such as the use of sunscreen, time of sun exposure, air pollution, food intake, levels of parathormone and calcium were not controlled in the analysis.

Another possible limitation is the time of interval between the cognitive impairment screening and the blood sample collection to measure 25(OH)D. The screening of cognitive impairment is a result of an ongoing process over years. On the other hand, 25(OH)D corresponds to a specific moment. Data suggests that the world is experiencing vitamin D insufficiency in a pandemic manner, pointing some risk factor as skin colour (with increased skin melanin pigmentation), obesity, less sun exposure, especially in children, pregnant and elderly population^(10)^. Also, decreased sun exposure associated with low consumption of foods containing vitamin D could be the main cause for this health issue^(80)^. Furthermore, the low monitoring of vitamin D deficiency in some countries and the poor health coverage for the population, could make it difficult to treat it early, contributing to perpetuate this scenario in the world^(10)^. In our study, only 55 older adults (∼10 % of the sample) reported the use of vitamin D supplements, despite the high prevalence of deficiency. In this way, if a high-risk population is not being monitored for 25(OH)D levels, it is possible to suggest that older adults in general could present vitamin D deficiency and are not receiving early treatment. This could partially explain the association between vitamin D deficiency and neuropsychiatric problems that we and other authors have been observed, considering that neuropsychiatric problems take some years to generate detectable symptoms.

Among the strengths, it is important to mention that the study has a probabilistic sampling, considering the distribution of the study population at the collection site. In addition, the use of validated and standardised instruments, training of the field team and quality control of the data performed in 10 % of the sample are factors that guaranteed the quality of the collected data. Furthermore, the adjustment for the season during which the blood sample was collected and for vitamin D supplementation are precautions that not all studies report.

## Conclusion

Our results suggest that the lowest quartile of vitamin D is independently associated with the probable presence of cognitive impairment in a sample of older people. This finding contributes to the understanding of the involvement of vitamin D in cognitive function and emphasises the importance of this micronutrient for the older adult population, as it appears to be a risk group for vitamin D deficiency. Considering the possible relationship between vitamin D and the mental health of older adults, more research is needed to investigate whether the maintenance of adequate serum concentrations could represent a significant factor in the prevention of age-related neurological disorders as well as to verify the need for new cutoff points for older adults concerning mental health.

## Supplementary material

For supplementary material accompanying this paper visit https://doi.org/10.1017/S1368980021004407. click here to view supplementary material
