# Supplementary material for: Association between lower serum vitamin D (25-hydroxy-cholecalciferol) concentrations and cognitive impairment in older adults: data from a populational-based cohort study in a middle-income country
Source: Public Health Nutr. 2021 Oct 25;25(9):2507–16. doi: 10.1017/S1368980021004407 (PMC9991716; doi:10.1017/S1368980021004407)
Supplement: Supplementary file 1 [file S1368980021004407sup001.docx]

**Supplementary Table** – Serum concentration of vitamin D of the elderly sample of the study according to the presence of cognitive impairment, EpiFloripa Aging cohort study, follow-up wave 2013-2014, Southern Brazil.

|  |  | |  | |  | | |
| --- | --- | --- | --- | --- | --- | --- | --- |
| **Characteristics (n=571)** | **Total** | | **Absent cognitive impairment** | | **Probable cognitive impairment** | | **p^1^** |
|  | **n** | **% (95%CI)** | **n** | **% (95%CI)** | **n** | **% (95%CI)** |  |
| **Endocrine Society** |  |  |  |  |  |  |  |
| Sufficiency (≥30ng/ml_ | 192 | 35.1 (29.1; 41.6) | 159 | 82.9 (75.2; 88.6) | 33 | 17.1 (11.4; 24.8) | 0.107 |
| Insufficiency (20-29 ng/ml) | 249 | 42.2 (36.8; 47.9) | 193 | 74.9 (66.7; 81.5) | 56 | 25.1 (18.5; 33.3) |  |
| Deficiency (<20 ng/ml) | 130 | 22.6 (17.3; 29.0) | 95 | 77.6 (66.2; 86.0) | 35 | 22.4 (14.0; 33.8) |  |
| **US Institute of Medicine** |  |  |  |  |  |  |  |
| Sufficiency (≥20ng/ml) | 441 | 77.4 (71.0; 82.7) | 352 | 78.5 (72.3; 83.6) | 89 | 21.5 (16.4; 27.7) | 0.050 |
| Insufficiency (12-20 ng/ml) | 99 | 17.9 (13.0; 24.2) | 76 | 81.0 (67.2; 89.9) | 23 | 19.0 (10.1; 32.8) |  |
| Deficiency (<12 ng/ml) | 31 | 4.7 (3.1; 7.2) | 19 | 64.7 (42.3; 82.1) | 12 | 35.3 (17.9; 57.7) |  |
| ^1^Chi-square | | | | | | | |
